# Supplementary material for: Assessment of Drought and Zinc Stress Tolerance of Novel Miscanthus Hybrids and Arundo donax Clones Using Physiological, Biochemical, and Morphological Traits
Source: Biology (Basel). 2023 Dec 14;12(12):1525. doi: 10.3390/biology12121525 (PMC10741058; doi:10.3390/biology12121525)
Supplement: Supplementary file 1 [file biology-12-01525-s001.zip › biology-2759949-supplementary.pdf]

biology

MDPI

**Table S1.** Estimated drought and Zn stress tolerance indices (stress tolerance indexes, STI) among the seven *Miscanthus* hybrids lines based on growth including; plant height (PH), number of leaves (NOL), shoot dry weight (SDW) , physiological; the maximum quantum efficiency of the PSII (Fv/Fm), performance index (PI-ABS), leaf relative water content (RWC),and biochmemical; protein, enzymes including polyphenol oxidase (PPO), peroxidase (POD), superoxide dismutase (SOD) and ascorbate peroxidase (APX), lipid peroxidation (MDA), parameters.

| ***Miscanthus*** | | | | | | | | | | | | | | |
|---|---|---|---|---|---|---|---|---|---|---|---|---|---|---|
| **Drought STI values** | | | | | | | | | | | | | | |
| **Hybrids** | **PH** | **NOL** | **SDW** | **Fv/Fm** | **PI** | **RWC%** | **Protein** | **PPO** | **POD** | **SOD** | **APX** | **MDA** | Phenol | Proline |
| GRC1 | 0.87 | 0.95 | 0.31 | 0.95 | 0.78 | 0.75 | 0.95 | 1.50 | 0.61 | 1.49 | 0.37 | 1.81 | 1.05 | 2.49 |
| GRC3 | 0.87 | 0.89 | 0.39 | 0.42 | 0.34 | 0.53 | 0.42 | 1.26 | 0.75 | 0.81 | 0.55 | 0.59 | 0.52 | 1.26 |
| GRC6 | 0.58 | 0.84 | 0.44 | 0.38 | 0.27 | 0.84 | 0.38 | 0.90 | 1.90 | 0.47 | 0.98 | 1.80 | 1.33 | 2.77 |
| GRC9 | 0.66 | 0.45 | 0.38 | 0.77 | 0.57 | 0.87 | 0.77 | 0.58 | 0.62 | 0.46 | 1.81 | 0.83 | 1.23 | 1.60 |
| GRC10 | 1.24 | 1.07 | 1.87 | 1.17 | 1.16 | 1.19 | 1.17 | 1.42 | 1.95 | 1.44 | 3.44 | 0.84 | 5.07 | 4.49 |
| GRC14 | 0.74 | 0.70 | 0.60 | 0.59 | 0.52 | 1.14 | 0.59 | 1.04 | 0.94 | 1.64 | 1.45 | 2.53 | 2.01 | 2.21 |
| GRC15 | 0.85 | 0.59 | 0.76 | 0.61 | 0.56 | 0.90 | 0.61 | 1.05 | 1.05 | 0.82 | 1.04 | 3.45 | 2.17 | 1.77 |
| **Zn STI values** | | | | | | | | | | | | | | |
| GRC1 | 0.90 | 1.06 | 0.46 | 0.85 | 0.72 | 0.66 | 0.63 | 1.34 | 0.69 | 2.55 | 0.42 | 2.29 | 1.00 | 2.63 |
| GRC3 | 0.82 | 0.85 | 0.33 | 0.46 | 0.44 | 0.54 | 1.14 | 1.06 | 0.78 | 0.88 | 0.55 | 0.73 | 0.63 | 1.25 |
| GRC6 | 0.64 | 0.87 | 0.49 | 0.75 | 0.49 | 0.86 | 1.19 | 1.12 | 2.17 | 0.60 | 1.07 | 1.84 | 1.50 | 2.31 |
| GRC9 | 0.79 | 0.40 | 0.34 | 0.45 | 0.31 | 0.91 | 0.79 | 0.65 | 0.65 | 0.52 | 1.68 | 1.32 | 1.32 | 1.60 |
| GRC10 | 1.25 | 1.07 | 1.49 | 1.19 | 1.01 | 1.21 | 1.82 | 1.58 | 1.84 | 1.54 | 3.42 | 0.94 | 4.34 | 3.77 |
| GRC14 | 0.75 | 0.61 | 0.64 | 0.41 | 0.35 | 1.00 | 1.19 | 1.10 | 0.94 | 1.75 | 1.59 | 3.08 | 2.18 | 2.48 |
| GRC15 | 0.89 | 0.59 | 0.69 | 0.44 | 0.37 | 0.76 | 1.35 | 1.06 | 1.02 | 0.81 | 1.02 | 3.67 | 2.23 | 1.62 |

**Table S2.** Estimated drought and Zn stress tolerance indices (stress tolerance indexes, STI) among the seven giant reed clones based on growth including; plant height (PH), number of leaves (NOL), shoot dry weight (SDW) , physiological; the maximum quantum efficiency of the PSII (Fv/Fm), performance index (PI-ABS), leaf relative water content (RWC),and biochmemical; protein, enzymes including polyphenol oxidase (PPO), peroxidase (POD), superoxide dismutase (SOD) and ascorbate peroxidase (APX), lipid peroxidation (MDA), parameters.

| **Giant reed** | | | | | | | | | | | | | | |
|---|---|---|---|---|---|---|---|---|---|---|---|---|---|---|
| **Drought STI values** | | | | | | | | | | | | | | |
| **Clones** | **PH** | **NOL** | **SDW** | **Fv/Fm** | **PI** | **RWC%** | **Protein** | **PPO** | **POD** | **SOD** | **APX** | **MDA** | **Phenol** | **Proline** |
| A1 | 0.83 | 0.77 | 0.55 | 0.81 | 0.67 | 0.84 | 0.49 | 1.12 | 2.21 | 0.35 | 0.46 | 2.38 | 0.50 | 1.68 |
| ASR | 0.68 | 0.67 | 0.25 | 0.88 | 0.08 | 0.85 | 1.28 | 1.66 | 0.94 | 2.10 | 1.13 | 0.83 | 0.72 | 1.99 |
| CT2 | 0.22 | 0.41 | 0.10 | 0.83 | 0.58 | 0.69 | 1.50 | 0.90 | 0.65 | 2.05 | 1.42 | 2.30 | 0.83 | 0.84 |
| PI1 | 0.40 | 0.62 | 0.30 | 0.62 | 0.44 | 0.74 | 1.15 | 0.88 | 0.96 | 0.76 | 1.21 | 1.82 | 1.53 | 1.51 |
| PC1 | 1.86 | 1.50 | 0.81 | 1.04 | 2.13 | 0.86 | 1.44 | 2.17 | 2.37 | 3.35 | 2.40 | 3.10 | 3.68 | 3.02 |
| PC6 | 0.79 | 0.75 | 0.37 | 0.97 | 0.88 | 0.72 | 0.67 | 1.27 | 0.94 | 0.87 | 0.61 | 3.02 | 1.15 | 1.36 |
| PC7 | 1.02 | 0.46 | 0.33 | 0.70 | 0.62 | 0.71 | 1.02 | 0.77 | 0.75 | 1.13 | 1.08 | 2.28 | 0.82 | 1.04 |
| **Zn STI values** | | | | | | | | | | | | | | |
| A1 | 0.91 | 0.90 | 0.52 | 0.88 | 0.56 | 0.87 | 0.53 | 1.16 | 2.07 | 0.35 | 0.52 | 2.06 | 0.50 | 1.65 |
| ASR | 0.68 | 0.67 | 0.25 | 0.84 | 0.06 | 0.87 | 1.30 | 1.41 | 1.19 | 1.90 | 1.33 | 1.22 | 0.76 | 2.03 |
| CT2 | 0.25 | 0.33 | 0.11 | 0.84 | 0.45 | 0.63 | 1.31 | 0.92 | 0.67 | 1.34 | 1.46 | 2.01 | 0.87 | 0.83 |
| PI1 | 0.36 | 0.62 | 0.27 | 0.51 | 0.78 | 0.81 | 1.23 | 0.88 | 0.82 | 1.07 | 1.30 | 1.25 | 1.65 | 1.53 |
| PC1 | 1.75 | 1.66 | 0.74 | 1.03 | 2.20 | 0.95 | 1.52 | 2.23 | 1.78 | 3.76 | 2.19 | 2.99 | 3.59 | 2.88 |
| PC6 | 0.92 | 0.82 | 0.39 | 1.00 | 0.80 | 0.83 | 0.82 | 1.27 | 1.11 | 1.26 | 0.90 | 2.84 | 1.11 | 1.67 |
| PC7 | 1.05 | 0.49 | 0.28 | 0.82 | 0.50 | 0.77 | 0.80 | 0.83 | 0.74 | 1.10 | 0.77 | 0.86 | 0.84 | 1.08 |

**Table S3.** Contributions and correlations of each growth and physiological parameter to the three main components of the PCA, used in the PCA ranking procedure based on the STI (drought and Zn) of *Miscanthus* hybrid lines and giant reed clones.

| ***Miscanthus* hybrids** | | | | | | | | | | | | |
|---|---|---|---|---|---|---|---|---|---|---|---|---|
| | **Drought** | | | | | | **Zn** | | | | | |
| | Contributions | | | Correlations | | | Contributions | | | Correlations | | |
| Parameters | PC1 | PC2 | PC3 | PC1 | PC2 | PC3 | PC1 | PC2 | PC3 | PC1 | PC2 | PC3 |
| PH | 8.98 | 11.66 | 0.02 | 0.82 | -0.54 | -0.02 | 8.20 | 2.09 | 1.69 | 0.84 | -0.22 | 0.17 |
| NOL | 6.42 | 13.65 | 1.61 | 0.69 | -0.59 | 0.17 | 5.81 | 18.53 | 0.03 | 0.71 | -0.64 | -0.02 |
| SDW | 11.81 | 0.13 | 2.98 | 0.94 | 0.06 | 0.23 | 10.45 | 0.77 | 1.50 | 0.95 | 0.13 | 0.16 |
| Fv/Fm | 7.51 | 0.06 | 21.99 | 0.75 | -0.04 | -0.62 | 8.83 | 7.03 | 0.51 | 0.87 | -0.40 | -0.09 |
| PI | 9.34 | 0.10 | 15.30 | 0.84 | -0.05 | -0.52 | 9.59 | 7.55 | 0.35 | 0.91 | -0.41 | -0.08 |
| RWC% | 6.99 | 15.69 | 0.15 | 0.72 | 0.63 | -0.05 | 6.39 | 12.60 | 1.21 | 0.74 | 0.53 | -0.14 |
| Protein | 3.50 | 12.00 | 22.70 | 0.51 | -0.55 | 0.63 | 8.46 | 1.55 | 0.03 | 0.85 | 0.19 | -0.02 |
| PPO | 4.85 | 15.12 | 0.00 | 0.60 | -0.62 | -0.01 | 10.85 | 2.71 | 0.19 | 0.96 | -0.25 | -0.06 |
| POD | 5.28 | 4.61 | 18.55 | 0.63 | 0.34 | 0.57 | 5.08 | 4.44 | 17.18 | 0.66 | 0.32 | -0.53 |
| SOD | 8.19 | 0.22 | 0.01 | 0.78 | -0.08 | -0.01 | 3.04 | 4.42 | 34.08 | 0.51 | -0.31 | 0.74 |
| APX | 6.96 | 11.66 | 0.28 | 0.72 | 0.54 | -0.07 | 8.26 | 10.02 | 0.50 | 0.84 | 0.47 | -0.09 |
| MDA | 0.54 | 5.60 | 15.62 | -0.20 | 0.38 | 0.53 | 2.87 | 5.87 | 28.29 | -0.50 | 0.36 | 0.68 |
| Phenol | 11.38 | 5.32 | 0.03 | 0.92 | 0.37 | 0.02 | 5.71 | 19.62 | 0.89 | 0.70 | 0.66 | 0.12 |
| Proline | 8.24 | 4.19 | 0.76 | 0.79 | 0.33 | 0.12 | 6.45 | 2.80 | 13.55 | 0.74 | 0.25 | 0.47 |
| **Giant reed clones** | | | | | | | | | | | | |
| | **Drought** | | | | | | **Zn** | | | | | |
| | Contributions | | | Correlations | | | Contributions | | | Correlations | | |
| Parameters | PC1 | PC2 | PC3 | PC1 | PC2 | PC3 | PC1 | PC2 | PC3 | PC1 | PC2 | PC3 |
| PH | 8.36 | 8.54 | 0.06 | 0.85 | -0.43 | -0.03 | 7.74 | 9.04 | 9.04 | 0.81 | -0.46 | -0.35 |
| NOL | 10.63 | 0.45 | 2.35 | 0.96 | -0.10 | 0.19 | 11.04 | 0.00 | 2.36 | 0.97 | -0.01 | -0.18 |
| SDW | 9.90 | 2.39 | 1.03 | 0.93 | -0.23 | 0.12 | 11.03 | 0.13 | 0.00 | 0.97 | -0.06 | 0.01 |
| Fv/Fm | 4.76 | 0.09 | 22.03 | 0.64 | -0.04 | -0.57 | 2.80 | 25.71 | 5.94 | 0.49 | -0.78 | 0.29 |
| PI | 6.50 | 6.26 | 6.24 | 0.75 | -0.37 | 0.30 | 7.64 | 1.92 | 0.30 | 0.81 | 0.21 | -0.06 |
| RWC% | 5.13 | 0.04 | 20.12 | 0.67 | -0.03 | -0.54 | 7.01 | 2.59 | 6.52 | 0.77 | -0.25 | -0.30 |
| Protein | 4.00 | 24.28 | 2.03 | 0.59 | 0.73 | 0.17 | 6.16 | 10.22 | 13.68 | 0.73 | 0.49 | 0.43 |
| PPO | 9.89 | 0.46 | 5.10 | 0.93 | 0.10 | -0.27 | 11.31 | 0.14 | 0.01 | 0.98 | -0.06 | -0.01 |
| POD | 10.55 | 0.00 | 3.61 | 0.96 | -0.01 | 0.23 | 10.65 | 0.34 | 0.34 | 0.95 | -0.09 | -0.07 |
| SOD | 1.61 | 22.24 | 8.56 | -0.37 | 0.70 | 0.35 | 0.02 | 33.20 | 7.35 | -0.04 | 0.88 | -0.32 |
| APX | 6.91 | 9.34 | 4.36 | 0.77 | 0.45 | 0.25 | 4.56 | 6.04 | 29.03 | 0.62 | 0.38 | 0.63 |
| MDA | 1.75 | 25.41 | 18.18 | -0.39 | -0.75 | 0.52 | 0.17 | 2.72 | 24.87 | 0.12 | -0.25 | 0.59 |
| Phenol | 9.38 | 0.49 | 6.25 | 0.90 | 0.10 | 0.30 | 8.82 | 7.60 | 0.25 | 0.87 | 0.42 | -0.06 |
| Proline | 10.65 | 0.01 | 0.07 | 0.96 | 0.01 | 0.03 | 11.05 | 0.33 | 0.31 | 0.97 | 0.09 | -0.07 |

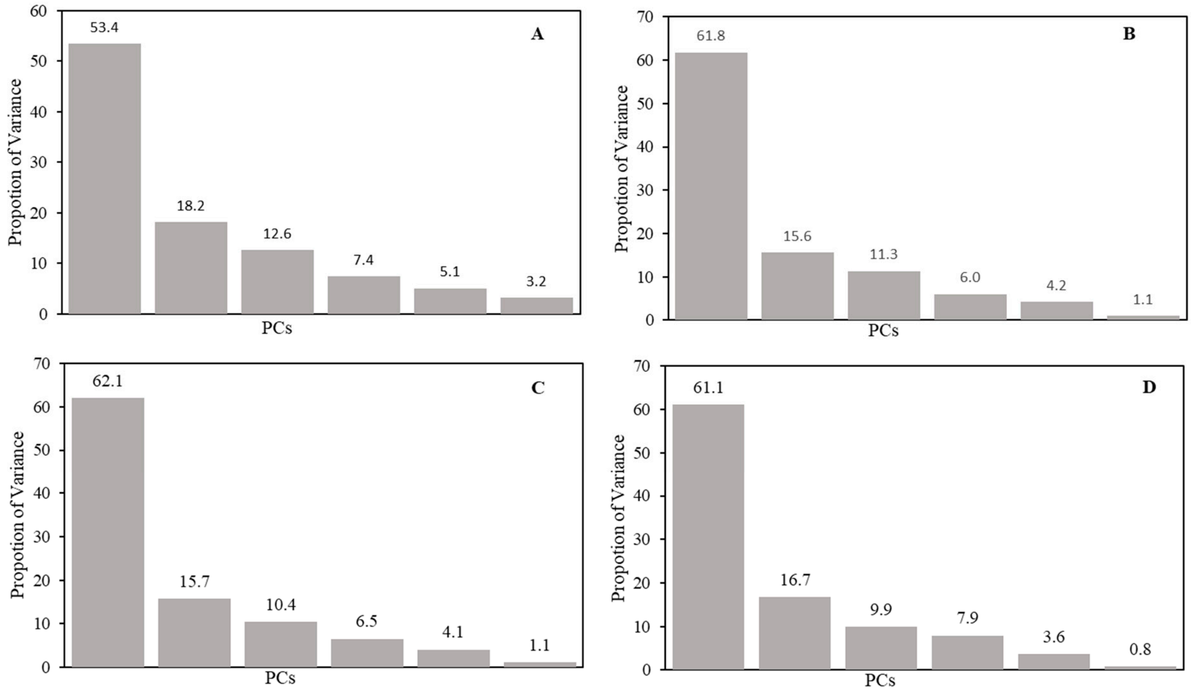


**Figure S1.** The proportion of variance for principal component analysis (PCA) based on the STI of growth and physiological traits of *Miscanthus* hybrids under drought **(A)** and under Zn **(B)** stress conditions and giant reed clones **(C)** and **(D)**, respectively.
